# Assessing the Quality of Life of Dogs with Inflammatory Bowel Disease and Their Owners

**DOI:** 10.3390/vetsci10070405

**Published:** 2023-06-21

**Authors:** David Díaz-Regañón, Ángel Sainz, Fernando Rodríguez-Franco, Alejandra Villaescusa, Patricia Olmeda, Ana Morcillo, Mercedes García-Sancho

**Affiliations:** Department of Animal Medicine and Surgery, College of Veterinary Medicine, Complutense University of Madrid, Avda. Puerta de Hierro s/n, 28040 Madrid, Spain; drdiazreganon@ucm.es (D.D.-R.); ferdiges@ucm.es (F.R.-F.); alejandrav@vet.ucm.es (A.V.); patolm01@ucm.es (P.O.); anamorci@ucm.es (A.M.); mercgarc@ucm.es (M.G.-S.)

**Keywords:** inflammatory bowel disease, chronic enteropathy, dog, quality of life, pet owner

## Abstract

**Simple Summary:**

Chronic diseases have been shown to affect the quality of life (QoL) of both humans and pets. Chronic gastrointestinal diseases, such as canine inflammatory bowel disease (IBD), are a common cause of chronic diarrhea, vomiting, and weight loss, affecting the normal daily lives of the dogs and their owners. In this study, we aimed to assess the associations between canine IBD and both dog and owner QoL, as well as the quality of the dog–owner relationship, using a survey. A total of 110 respondents (30 owners of dogs with IBD and 80 owners of healthy dogs) completed the questionnaire. Dogs with IBD presented a lower overall QoL, health status, and activity levels. Owners of dogs with IBD had lower QoL and more negative impact on their QoL, more daily limitations due to their dog’s disease, and more distress compared to owners of healthy dogs. In addition, IBD dog owners were more likely to consider their dogs as their children. Regardless of the severity of the disease, IBD has a detrimental effect on affected dogs and their owners.

**Abstract:**

The aim of the study was to assess the quality of life (QoL) of dogs with inflammatory bowel disease (IBD) and the impact on the life and relationship of the owner. An online questionnaire based on a Likert scale score (1–10) was designed to assess items related to dog QoL, owner QoL, and the owner–dog relationship. Responses from 110 dog owners (30 with IBD and 80 healthy dogs) were included in the study. IBD dogs had significantly lower overall QoL (*p* < 0.001), health (*p* < 0.0001), and level of activity (*p* = 0.049). Owners of dogs with IBD reported lower overall QoL (*p* < 0.001). The scores for how their dog’s QoL might affect their own QoL (*p* = 0.028), how much their dog limited their social life, leisure time, or daily activities (*p* = 0.015), and how often they felt burdened by caring for their dog (*p* = 0.006) were significantly higher in the IBD group when compared to the healthy group. In addition, IBD dog owners were more likely to see their dogs as children (*p* = 0.0004). IBD has a negative impact on affected dogs and their owners regardless of the severity of the disease.

## 1. Introduction

Quality of life (QoL) is a challenging concept of subjective nature that encompasses a multifaceted assessment of physical, psychological, and social well-being [1,2]. However, this term lacks an overall definition and a defined approach to measurement [2].

Similar to human medicine, the assessment of QoL constitutes an important and increasingly common outcome measure in veterinary research and clinical practice, particularly in dogs [2,3]. This evaluation has been shown to be useful in providing comprehensive care and guiding treatment decisions [2]. 

In veterinary medicine, all tools used to evaluate QoL are classified as observed-reported outcomes (OROs) as animals cannot verbally express how they feel [4]. Although some assessment tools have been developed to assess the QoL of healthy dogs [3], most QoL assessment tools are disease-specific and have been described to evaluate the QoL of dogs with skin diseases [5,6,7,8], cardiac diseases [9], epilepsy [10,11,12,13], obesity [14,15], Cushing’s syndrome [16], or cancer [17].

Despite gastrointestinal signs being one of the most common reasons for veterinary visits [18,19], the QoL of dogs with chronic gastrointestinal diseases has received little attention in veterinary research. To the authors’ knowledge, only one recent study has implemented a QoL assessment tool for dogs with chronic enteropathies [20], and another previous study considered the assessment of general QoL in the follow-up of canine inflammatory bowel disease (IBD) [21]. 

Numerous studies in human medicine have shown that the QoL of IBD patients deteriorates [22,23,24]. However, it is well known that human IBD not only affects the QoL of the patients but also the well-being of their family members [25,26]. The human-dog bond has stood the test of time, evolving from a historic partnership rooted in work and security to a deep emotional connection. As a result, dogs have become integral members of the family for most of the owners [27]. However, recognizing that the well-being of dogs is closely intertwined with that of their human counterparts is a relatively new approach that has received considerable attention [28]. 

Therefore, the aim of this research was to assess the impact of canine IBD not only on the quality of life of dogs but also on their owners and the impact on the owner–dog relationship, using a designed and validated QoL assessment survey. 

## 2. Materials and Methods

This retrospective study was conducted at the Complutense Veterinary Teaching Hospital (CVTH) of the Complutense University of Madrid (UCM), Spain. A favorable opinion on this study was given by the Committee on Bioethics of the UCM (Review No. CE_20221110-02_SAL).

### 2.1. Questionnaire Design and Distribution

The information was analyzed anonymously and confidentially. The questionnaire was designed based on previously validated surveys assessing the QoL in dogs with various chronic diseases and adapted to canine IBD [9,10,20]. Owners of dogs with and without IBD and veterinarians used the questionnaire as a pilot to identify any unclear questions that needed revision. A final version of the updated questionnaire was distributed online (Google Forms) and by telephone using a non-probabilistic sampling and consisted of multiple sections. The items were scored on a Likert scale from 1 to 10, as shown below. The sections were:

- Dog signalment shall include age (years), sex (female or male), body weight (kg), and breed;
- Dog QoL items (at the time of the diagnosis if they belonged to the IBD group) were scored according to the visual scale for the assessment of the QoL described by Marchetti et al., [20]:
  1. General QoL (1: very poor; 10: very good);
  2. Health status (1: very ill; 10: not ill);
  3. Activity level (1: apathetic/lethargic; 10: active);
  4. Interaction with owners or family (1: deteriorated; 10: very good);
  5. Level of stimulation (e.g., walks, play, training) (1: none; 10: many);
- Owner demographics including age (years) and gender (woman or man);
- Owner QoL items include:
  1. General QoL (1: very poor; 10: very good);
  2. Owner’s QoL affected by the dog’s QoL (1: none; 10: too much);
  3. Life limitation of the owner by the dog (1: never; 10: always);
  4. How often owner is the owner burdened by caring for the dog (1: never; 10: always);
- Owner–dog relationship includes:
  1. Considering the dog as a child (1: none; 10: too much);
  2. The dog understands the owner’s mood or problems (1: none; 10: too much);
  3. Feeling closer to the dog than to friends or family members (1: none; 10: too much);
- Impact of the disease (only in the group of respondents with IBD dogs):
  1. Impact of IBD on owner´s QoL (1: negative; 10: positive);
  2. Care since the diagnosis changed the relationship (1: weaker; 10: stronger).

In addition, body condition score (BCS; 1–9) and canine chronic enteropathy clinical activity index (CCECAI) [29] scores were obtained from the clinical records of these IBD dogs at the time of the diagnosis. The CCECAI index score was categorized as low (insignificant or mild disease; 0–5) and high (moderate or severe disease; ≥6). In the case of dogs with IBD, all the survey questions related specifically to the time of diagnosis. (You can find an English or Spanish version of the questionnaire in the Supplementary Materials.)

### 2.2. Respondent and Dog Inclusion

Participation in the study was voluntary. Owners in the IBD group were recruited from cases diagnosed at the CVTH Gastroenterology and Endoscopy service (*n* = 30). The IBD patients were diagnosed at CVMTH by the Gastroenterology and Endoscopy service based on the World Small Animal Veterinary Association (WSAVA) criteria [30]. The diagnostic protocol included a complete physical examination, complete blood count, serum biochemistry panel, IFA test for *Leishmania infantum* and *Ehrlichia canis*, direct and indirect fecal examination for nematode and protozoan parasite detection, TLI (trypsin-like immunoreactivity), resting cortisol/ACTH stimulation test, and diagnostic imaging (abdominal ultrasound and/or radiographs). In addition, the dogs showed an insufficient response to dietary modification alone, but responded to immunosuppressive treatment. 

The healthy control (HC) group consisted of 80 dogs recruited openly from the general public. The owners indicated that the dog was healthy by answering a specific first question on the questionnaire. In addition, a second question was completed to determine if the dog had any concurrent diseases or clinical signs. Dogs less than 2 years of age or with any concurrent disease or clinical sign were excluded from the study. 

### 2.3. Statistical Analysis

The data obtained were statistically analyzed using the software SAS, version 9.4 (SAS Institute, Cary, NC, USA). The Shapiro–Wilk test was performed to assess the normal distribution of the data. Comparisons between groups with respect to the signalment of the dogs and owner age and sex were made using the Student T-test for numerical variables and the Chi-square test for categorical variables. The median scores of the questionnaire items relating to dog QoL, owner QoL, and their relationship depending on the group (HC vs. IBD) were compared using the Wilcoxon rank-sum test. The Spearman correlation coefficient was used to assess possible correlations between variables. The significance level was set at *p* < 0.05. The internal consistency of the owner QoL questionnaire was validated using Cronbach’s alpha (Cronbach’s α = 0.7). Cronbach’s α = 0.7–0.9 corresponds to an adequate homogeneity of the items.

## 3. Results

### 3.1. Demographics

When analyzing the dogs’ signalment, there were no significant differences between the IBD and healthy control groups of dogs in terms of age, weight, sex, or breed. There were also no significant differences in the age or gender of the owner respondents between groups (Table 1).

### 3.2. QoL of Dogs

The QoL scores of the dogs in the IBD group were lower than those of the healthy dogs for all items assessed. The differences were statistically significant for overall QoL, health status, and activity level of the dog (Table 2). 

In dogs with IBD, no statistical differences were found when analyzing these items based on the sex or the breed of the dog. However, there was a negative correlation between the age of the dogs and items related to activity level (r = −0.424; *p* = 0.019) and stimulation (r = −0.432; *p* = 0.017). When the QoL variables of the dogs were compared according to the gender of the owner, women gave higher scores to the interaction (*p* = 0.003) and to the stimulation of their dogs (*p* = 0.039) compared to men. 

Most of the diseased animals (75%; *n* = 21/28) had a high disease activity index compared to those with a low CCECAI score (25%; *n* = 7/28). When comparing dogs with a clinically insignificant or mild disease to those with moderate-to-very severe IBD (CCECAI ≥ 6), no differences were observed in the canine QoL items (Table 3). Similarly, no correlation was found between CCECAI score and any of the five canine QoL items. The median BCS of IBD dogs was 4 (mean 4.45 ± 1.45, range 2 to 8).

### 3.3. QoL of Owners

Regarding the owner QoL items, the score assigned to general QoL was significantly lower in the IBD dog owners. On the contrary, the scores for how their dogs’ QoL might affect their own QoL, how much their dog limited their social life, leisure time, or daily activities, and how often they felt overwhelmed or burdened by caring for their dogs were significantly higher in the IBD group (Table 4). No statistical differences were found when analyzing these items based on the gender or the age of the owners. 

In addition, a moderately positive correlation was observed between the general QoL of the owner and that of the IBD dog (r = 0.408; *p* = 0.025).

When the IBD dog owners were asked about the impact that their pet’s disease had had on their own QoL (1: negative impact to 10: positive impact), the median score was 4 out of 10 (3.97 ± 2.36). This impact did not correlate with disease severity as measured by the CCECAI score (r = −0.189; *p* = 0.334). In terms of how caring for their pet had changed their relationship since the diagnosis (1: weaker to 10: stronger), a median score of 8 was obtained (7.37 ± 2.22). 

### 3.4. Dog–Owner Relationship 

The scores for the three items assessing the relationship between the owner and their dog were higher in the IBD group than in the healthy group, with the score for whether they considered their dog to be a child being statistically significantly higher (Table 5). The gender or age of the owners did not show any statistically differences in the analysis of these items.

The assessment of this perception as a child was negatively correlated with general QoL of the owner (r = -0.454; *p* = 0.012) and positively correlated with the limitation that their dog imposed on their own social life, free time, or daily activities (r = 0.385; *p* = 0.035). The closeness to the dog score was positively correlated with the scores assessing how their dogs’ QoL could affect their own QoL (r = 0.400; *p* = 0.028) and the limitations on the owner’s life (r = 0.369; *p* = 0.045). In addition, no correlation was found between items related to the dog–owner relationship and the dogs’ QoL.

## 4. Discussion

This study firstly documented the significant impact of canine IBD on both dogs’ and owners’ QoL, outlining a connection between them.

As expected, dogs with IBD had a lower QoL than healthy dogs for all variables. In support of this information, Marchetti et al. reported similar results in dogs with chronic enteropathies such as food-responsive, antibiotic-responsive, immunosuppressant-responsive, and protein-losing enteropathies [20]. Regarding exclusively IBD, there is only one previous study in which the owners rated the dog’s QoL from 1 to 10 at the time of diagnosis and at follow-up [21]. In this study by Craven et al., owner-evaluated QoL at follow-up was significantly associated with outcome, suggesting that owner perceptions are a valid aspect of disease monitoring.

It is recommended that appropriate and validated instruments should be used to assess canine QoL and that the use of novel, unvalidated instruments should be avoided [31]. Based on this recommendation, the 1-10 visual scale developed specifically for enterophatic dogs was used in this study [20]. Items related to health, activity, interaction, and stimulation were also included in the Marchetti’s tool. A recent review suggested that activity and interaction are valuable QoL parameters to assess, but also that evaluation of appetite is important for both dogs and cats [2]. The dog would be less motivated to interact with the owner in the same way if it was not driven by food. Therefore, changes in dog–owner interactions could be reasonably expected when changes in a dog’s health changes lead to a decrease in appetite [17]. Future research should consider the potential impact of appetite on the QoL of dogs with chronic enteropathies such as IBD. 

While the sex of the dogs and the breed did not influence the QoL assessments, the age was associated with the level of activity and stimulation of the dogs. The older the age, the lower the activity and stimulation score. Although activity level and the desire for interaction are common items on generic QoL assessment tools in dogs [2], little is known about the potential effects of aging. A recent study assessing the health-related quality of life (HRQL) in healthy dogs found that older dogs experienced a general but mild decline in their energy, happiness, and activity levels [32]. Furthermore, in both humans and dogs, aging is generally associated with an increased number of chronic health problems that contribute to poorer QoL [10]. In contrast, this effect has not been described in dogs with Cushing’s syndrome [16]. Breed and sex predicted very little of the variation seen in HRQL scores seen in healthy dogs [32]. Surprisingly, the relationship between all these factors and QoL in dogs with chronic disease has not been well studied. 

The QoL of the dogs was not affected by the age of the owners as has been previously documented [8,9]. On the contrary, in the study by Favrot et al., older owners reported a worse QoL than younger owners. One possible explanation is that many older people might spend more time with their pet, and therefore have a stronger perception of the dog’s illness [6]. Therefore, it seems unclear whether the age of the owners should be taken into account when assessing the QoL of the dogs.

Our results showed a difference in gender perceptions of the dog’s QoL. Female owners rated interaction and stimulation items higher than male owners. This could be due to a possible more active involvement in the emotional care of their dog, which improves their interaction and the realization of joint activities such as walks and games. Similar to previous work, the overrepresentation of Caucasian females could introduce a bias in that these individuals may experience these items differently from other groups [33]. This is not the first time that differences between genders have been suggested, for example, in perceived stress levels in dogs [34]. In contrast, other authors reported that the gender of the owner had no effect on the dogs’ QoL [6,8,9]. Similarly, the educational level of the owners did not influence the perception of the disease [8].

No association between clinical severity and QoL was found in our dogs. This rather unexpected result is not consistent with previous studies evaluating different chronic disorders [8,9,16]. Specifically, in chronic enteropathies Marchetti et al. suggest that the more severe the enteropathy, the lower the dog’s QoL [20]. In humans with IBD the relationship between disease severity and QoL has been described [26,35]. However, in other areas of human medicine this relationship is often contradictory [7]. It could be argued that a strong association between clinical index and QoL measures raises the possibility that both instruments are assessing the same components of disease activity rather than the QoL measure reflecting additional factors. However, it is important to consider that the lack of association between clinical severity and QoL may also indicate the influence of other important factors, such as the implementation of treatments (e.g., medications or dietary trials), which may independently influence QoL outcomes. These additional interventions could potentially contribute to a decrease in QoL, even in cases with less severe clinical presentations. Thus, our findings support the use of QoL tools as they provide complementary data.

Having a dog with IBD has a negative effect on the QoL of its owner. In addition to this effect, dog and owner QoL were correlated. Similar results have been reported in an isolated study about idiopathic epilepsy in dogs [11]. However, this is the first time this situation has been described in canine IBD, although the impact of human IBD on family members is well known [25,26]. Families are the first source of daily support for most patients. In veterinary medicine this role is developed by the owner, so it is expected that both could experience relatively similar outcomes. Furthermore, it is notably accepted that pets are increasingly becoming members of the family, deserving a similar level of medical care as human family members receive, and are an essential part of many people’s daily lives [12,28].

As previously found by Spitznagel et al., owners of pets with chronic or terminal diseases reported greater burden, stress, and symptoms of depression and anxiety, as well as lower QoL [36,37]. Family carers of IBD patients were also more likely to suffer emotional problems and lower mental health scores compared to the general population [23]. IBD also affects family relationships with the patient, social life, daily activities, work, finances, leisure time, and travel [25,26]. Our owners of dogs with IBD experienced similar feelings, confirming that caring for a dog with IBD negatively affects not only their QoL but also their social life, leisure time, daily activities, and feelings of burden. Similar results have been described in dogs with other diseases such as epilepsy [10] or cardiac disease [9]. This supports the potential overlap between the roles of owners and family carers, as well as the importance of assessing owners’ QoL independently of the dogs’ QoL.

Family members of patients with IBD suggested that steps could be taken to improve their QoL, including better information about the disease and better access to a counsellor or psychologist [25]. This is something that clinicians should consider when communicating with owners. For example, a detailed explanation of canine IBD and what they can expect may be helpful in reducing the impact on their own QoL. On the other hand, knowledge of the client’s emotional state may help the veterinarian to respond empathetically and communicate effectively with a distressed patient in this situation. In addition to the clinical severity of the disease, the owner’s physical and psychological distress, and the social aspects of their dog’s condition, should be considered [8].

The impact the pet’s disease had on the owners’ QoL was not related to the severity of the disease. However, QoL scores were lower for owners of dogs with severe or poorly controlled epilepsy [13]. Favrot et al. also suggested that the more severe the atopic dermatitis, the greater the impact on the owner’s daily life [6]. It could be argued that each condition has specific characteristics and therefore the comparison between them is not accurate. For example, in the case of dogs with IBD, a clinically severe disease may be due to the presence of watery diarrhea, increased stool frequency, slightly decreased activity or appetite, moderate vomiting, and weight loss. These chronic features do not necessarily imply a severe impact on the owners’ QoL.

The current study also looked at the impact of IBD on the dog–owner relationship. Comparing dogs with IBD to healthy dogs, we found that owners of dogs with IBD were more likely to report considering their dog as a child than owners of healthy dogs. In addition, owners of dogs with IBD expressed how caring for their sick pet had strengthened their relationship. Previous studies of dogs with chronic enteropathies [20], heart disease [9] or skin disease [6] showed similar results. Among the three questions that assessed the relationship between the owner and their dog, treating the dog as a child stands out. More than 70% of owners of epileptic dogs reported treating their pet like a child [10]. The human-animal bond theory, which advocates animals as friends and family members, provides an explanation for this circumstance [28]. 

Furthermore, the literature suggests that the higher the level of owner attachment to the dog, the higher the perceived impact of their pet’s disease on their QoL and the lower their own QoL [6]. Therefore, including questions about the level of attachment between the dog and the owner may provide insightful data that will further our understanding of the QoL of IBD dog owners. Finally, as previously reported by Favrot et al. [6], the owner’s level of attachment does not influence their perception of the dog’s QoL. Although a dog owner with a dog that sleeps on their bed may not have the same feelings as a dog owner with a dog that lives in an outdoor run, both are able to assess the overall QoL of their pets, as well as health, activity, interaction, and stimulation factors. Veterinarians should be aware that a closer bond between dog and owner does not always translate into a better ability of the owners to serve as proxy respondents when assessing the quality of life of their pets. In addition, recognizing the potential importance of human-animal support services, such as financial assistance programmes or the involvement of veterinary social workers, can play a crucial role in maintaining the human-animal bond. These services can address the unique needs of both owners and dogs with chronic conditions, providing essential support and promoting the overall well-being of both parties.

The current study has several limitations. Retrospective studies may be subject to recall bias, as owners would have to recall the information. This feature, however, may be controversial as some authors have found no associations between time since diagnosis and QoL scores [38]. Another limitation may be the lack of information on the treatments received by the dogs with IBD. It is known that the therapeutic interventions required to keep the pet’s dermatological diseases under control had a negative impact on the owners’ and the dogs’ QoL [5], although the monthly cost of medication was not associated with the QoL of owners of dogs with epilepsy [13]. However, the possible influence of these factors (such as the financial burden of treatment) in dogs with IBD is not known. Finally, no questions were included about the lifestyle of the dogs. In this sense, it has been previously observed that dogs living in rural or suburban areas have higher QoL [39]. An assessment of the dog’s environment might be useful to better understand the influence on the QoL of dogs with IBD and their owners.

## 5. Conclusions

Regardless of the severity of the disease, IBD not only affects the dog’s QoL but is also strongly associated with the owner’s QoL. Veterinarians should be aware that assessing the clinical severity of this disease does not always reflect the QoL of their patients and their owners. Therefore, assessment of QoL should be incorporated into clinical practice as it provides essential complementary information. Clinicians should also consider the higher risk of caregiver burden or psychosocial functioning problems for owners of dogs with IBD. Understanding the emotional state of the owner and providing better information about IBD may improve the veterinary support for these owners. This study provides a reference point for further research into QoL and IBD in dogs.

## Figures and Tables

**Table 1:** Epidemiological data of the dogs and their owners.

	HC (*n* = 80)	IBD (*n* = 30)	*p*-Value
Dog			
Age (years; mean ± SD)	6.78 ± 3.10	7.65 ± 3.53	0.207
BW (kg; mean ± SD)	20.98 ± 13.57	19.58 ± 13.20	0.628
Sex (female/male)	46/34	16/14	0.695
Breed (pure/mixed)	52/28	19/11	0.871
Owner			
Age (years; mean ± SD)	41.75 ± 11.63	45.90 ± 12.16	0.102
Gender (woman/man)	57/23	22/8	0.829

BW: Body weight; HC: Healthy control; IBD: Inflammatory bowel disease; SD: Standard deviation.

**Table 2:** Results of the dogs’ QoL items based on the condition (HC vs. IBD) of the dogs.

Items	HC (*n* = 80)	IBD (*n* = 30)	*p*-Value
Dog-QoL	Mean ± SD/Median (range)		
General QoL	9.10 ± 1.05/9 (6–10)	6.30 ± 2.55/7 (1–10)	<0.0001 *
Health	8.95 ± 1.18/9 (5–10)	6.07 ± 2.30/6 (2–10)	<0.0001 *
Activity	8.64 ± 1.62/9 (2–10)	7.63 ± 2.34/8.5 (2–10)	0.049 *
Interaction	9.41 ± 1.32/10 (1–10)	9.00 ± 1.72/10 (4–10)	0.404
Stimulation	8.29 ± 1.41/8 (4–10)	7.60 ± 2.30/8 (3–10)	0.341

HC: Healthy control; IBD: Inflammatory bowel disease; QoL: Quality of Life; SD: Standard deviation; * *p*-Value < 0.05.

**Table 3:** Results of the dogs’ QoL scores in IBD dogs based on CCECAI.

IBD-Dogs	Low CCECAI 0–5 (*n* = 7)	High CCECAI ≥6 (*n* = 21)	*p*-Value
Dog QoL items	Mean ± SD/Median (range)		
General QoL	6.86 ± 2.27/7 (4–9)	5.90 ± 2.62/7 (1–10)	0.369
Health	7.29 ± 2.14/8 (4–10)	5.86 ± 2.22/6 (2–10)	0.167
Activity	8.57 ± 1.51/9 (6–10)	7.57 ± 2.31/8 (3–10)	0.365
Interaction	9.29 ± 1.50/10 (6–10)	8.90 ± 1.87/10 (4–10)	0.751
Stimulation	7.71 ± 1.89/8 (5–10)	7.86 ± 2.31/8 (3–10)	0.667

CCECAI: Canine Chronic Enteropathy Clinical Activity Index; IBD: Inflammatory bowel disease; QoL: Quality of Life; SD: Standard deviation.

**Table 4:** Results of the owners’ QoL scores based on the condition of the dogs.

Items	HC (*n* = 80)	IBD (*n* = 30)	*p*-Value
Owner-QoL	Mean ± SD/Median (range)		
General QoL	8.39 ± 1.08/8 (5–10)	7.20 ± 1.90/7.50 (1–10)	0.001 *
Affected	8.04 ± 2.68/9 (1–10)	9 ± 1.88/10 (4–10)	0.028 *
Limitation	5.28 ± 2.81/5 (1–10)	6.77 ± 2.65/7 (2–10)	0.015 *
Burdened	3.63 ± 2.58/3 (1–10)	5.40 ± 2.99/5 (1–10)	0.006 *

HC: Healthy control; IBD: Inflammatory bowel disease; QoL: Quality of Life; SD: Standard deviation; * *p*-Value < 0.05.

**Table 5:** Results of the dog–owner relationship based on the condition (HC vs. IBD) of the dogs.

Items	HC (*n* = 80)	IBD (*n* = 30)	*p*-Value
Dog–owner relationship	Mean ± SD/Median (range)		
Child	6.76 ± 3.13/8 (1–10)	8.83 ± 2.02/10 (1–10)	0.0004 *
Mood	7.90 ± 2.05/8.50 (1–10)	8.33 ± 1.99/9 (3–10)	0.225
Closeness	6.46 ± 2.82/7 (1–10)	7.63 ± 1.92/8 (3–10)	0.087

HC: Healthy control; IBD: Inflammatory bowel disease; QoL: Quality of Life; SD: Standard deviation; * *p*-Value < 0.05.

## Data Availability

All the information obtained in this study is already included in the manuscript.

## Supplementary Materials

The following supporting information can be downloaded at: https://www.mdpi.com/article/10.3390/vetsci10070405/s1: English and Spanish versions of the questionnaire about quality of life.

## References

[1] The WHOQOL Group (1995). The World Health Organization quality of life assessment (WHOQOL): Position paper from the World Health Organization. Soc. Sci. Med..

[2] Fulmer A.E., Laven L.J., Hill K.E. (2022). Quality of Life Measurement in Dogs and Cats: A Scoping Review of Generic Tools. Animals.

[3] Lavan R.P. (2013). Development and validation of a survey for quality of life assessment by owners of healthy dogs. Vet. J..

[4] McMillan F.D. (2003). Maximizing quality of life in III animals. J. Am. Anim. Hosp. Assoc..

[5] Noli C. (2019). Assessing Quality of Life for Pets with Dermatologic Disease and Their Owners. Vet. Clin. N. Am. Small Anim. Pract..

[6] Favrot C., Linek M., Mueller R., Zini E., International Task Force on Canine Atopic Dermatitis (2010). Development of a questionnaire to assess the impact of atopic dermatitis on health-related quality of life of affected dogs and their owners. Vet. Dermatol..

[7] Noli C., Minafò G., Galzerano M. (2011). Quality of life of dogs with skin diseases and their owners. Part 1: Development and validation of a questionnaire. Vet. Dermatol..

[8] Noli C., Colombo S., Cornegliani L., Ghibaudo G., Persico P., Vercelli A., Galzerano M. (2011). Quality of life of dogs with skin disease and of their owners. Part 2: Administration of a questionnaire in various skin diseases and correlation to efficacy of therapy. Vet. Dermatol..

[9] Freeman L.M., Rush J.E., Clark M.A., Bulmer B.J. (2020). Validation and preliminary data from a health-related quality of life questionnaire for owners of dogs with cardiac disease. J. Vet. Intern. Med..

[10] Masucci M., Di Stefano V., Donato G., Mangano C., De Majo M. (2021). How Owners of Epileptic Dogs Living in Italy Evaluate Their Quality of Life and That of Their Pet: A Survey Study. Vet. Sci..

[11] Wessmann A., Volk H., Packer R., Ortega M., Anderson T. (2016). Quality-of-life aspects in idiopathic epilepsy in dogs. Vet. Rec..

[12] Rundfeldt C. (2016). Quality of life of dogs with chronic epilepsy. Vet. Rec..

[13] Nettifee J.A., Munana K.R., Griffith E.H. (2017). Evaluation of the Impacts of Epilepsy in Dogs on Their Caregivers. J. Am. Anim. Hosp. Assoc..

[14] Endenburg N., Soontararak S., Charoensuk C., van Lith H.A. (2018). Quality of life and owner attitude to dog overweight and obesity in Thailand and the Netherlands. BMC Vet. Res..

[15] Yam P., Butowski C., Chitty J., Naughton G., Wiseman-Orr M., Parkin T., Reid J. (2016). Impact of canine overweight and obesity on health-related quality of life. Prev. Vet. Med..

[16] Schofield I., O′Neill D.G., Brodbelt D.C., Church D.B., Geddes R.F., Niessen S.J. (2019). Development and evaluation of a health-related quality-of-life tool for dogs with Cushing′s syndrome. J. Vet. Intern. Med..

[17] Giuffrida M.A., Brown D.C., Ellenberg S.S., Farrar J.T. (2018). Development and psychometric testing of the Canine Owner-Reported Quality of Life questionnaire, an instrument designed to measure quality of life in dogs with cancer. J. Am. Vet. Med. Assoc..

[18] O’Neill D.G., James H., Brodbelt D.C., Church D.B., Pegram C. (2021). Prevalence of commonly diagnosed disorders in UK dogs under primary veterinary care: Results and applications. BMC Vet. Res..

[19] Jergens A.E., Heilmann R.M. (2022). Canine chronic enteropathy—Current state-of-the-art and emerging concepts. Front. Vet. Sci..

[20] Marchetti V., Gori E., Mariotti V., Gazzano A., Mariti C. (2021). The Impact of Chronic Inflammatory Enteropathy on Dogs’ Quality of Life and Dog-Owner Relationship. Vet. Sci..

[21] Craven M., Simpson J., Ridyard A., Chandler M. (2004). Canine inflammatory bowel disease: Retrospective analysis of diagnosis and outcome in 80 cases (1995–2002). J. Small Anim. Pract..

[22] Fourie S., Jackson D., Aveyard H. (2018). Living with Inflammatory Bowel Disease: A review of qualitative research studies. Int. J. Nurs. Stud..

[23] Liu R., Tang A., Wang X., Shen S. (2018). Assessment of Quality of Life in Chinese Patients with Inflammatory Bowel Disease and their Caregivers. Inflamm. Bowel Dis..

[24] van der Have M.V., van der Aalst K.S., Kaptein A.A., Leenders M., Siersema P.D., Oldenburg B., Fidder H. (2014). Determinants of health-related quality of life in Crohn′s disease: A systematic review and meta-Analysis. J. Chohns Colitis..

[25] Thapwong P., Norton C., Rowland E., Farah N., Czuber-Dochan W. (2023). A systematic review of the impact of inflammatory bowel disease (IBD) on family members. J. Clin. Nurs..

[26] Klomberg R.C., Aardoom M.A., Kemos P., Rizopoulos D., Ruemmele F.M., Croft N.M., de Ridder L., Neyt M., Turner D., Focht G. (2022). High Impact of Pediatric Inflammatory Bowel Disease on Caregivers’ Work Productivity and Daily Activities: An International Prospective Study. J. Pediatr..

[27] Cohen S.P. (2002). Can pets function as family members?. West. J. Nurs. Res..

[28] Scoresby K.J., Strand E.B., Ng Z., Brown K.C., Stilz C.R., Strobel K., Barroso C.S., Souza M. (2021). Pet Ownership and Quality of Life: A Systematic Review of the Literature. Vet. Sci..

[29] Washabau R.J., Day M.J., Willard M.D., Hall E.J., Jergens A.E., Mansell J., Minami T., Bilzer T.W., Group, WSAVA International Gastrointestinal Standardization Group (2010). Endoscopic, biopsy, and histopathologic guidelines for the evaluation of gastrointestinal inflammation in companion animals. J. Vet. Intern. Med..

[30] Allenspach K., Wieland B., Gröne A., Gaschen F. (2007). Chronic enteropathies in dogs: Evaluation of risk factors for negative outcome. J. Vet. Intern. Med..

[31] Belshaw Z., Yeates J. (2018). Assessment of quality of life and chronic pain in dogs. Vet. J..

[32] Rodger S., Scott E.M., Nolan A., Wright A.K., Reid J. (2021). Effect of Age, Breed, and Sex on the Health-Related Quality of Life of Owner Assessed Healthy Dogs. Front. Vet. Sci..

[33] Spitznagel M.B., Mueller M.K., Fraychak T., Hoffman A.M., Carlson M.D. (2019). Validation of an abbreviated instrument to assess veterinary client caregiver burden. J. Vet. Intern. Med..

[34] Mariti C., Gazzano A., Moore J.L., Baragli P., Chelli L., Sighieri C. (2012). Perception of dogs’ stress by their owners. J. Vet. Behav..

[35] Jowett S.L., Seal C.J., Barton J.R., Welfare M.R. (2001). The short inflammatory bowel disease questionnaire is reliable and responsive to clinically important change in ulcerative colitis. Am. J. Gastroenterol..

[36] Spitznagel M.B., Jacobson D.M., Cox M.D., Carlson M.D. (2017). Caregiver burden in owners of a sick companion animal: A cross-sectional observational study. Vet. Rec..

[37] Spitznagel M.B., Anderson J.R., Marchitelli B., Sislak M.D., Bibbo J., Carlson M.D. (2021). Owner quality of life, caregiver burden and anticipatory grief: How they differ, why it matters. Vet. Rec..

[38] Tzannes S., Hammond M.F., Murphy S., Sparkes A., Blackwood L. (2008). Owners ‘perception of their cats’ quality of life during COP chemotherapy for lymphoma. J. Feline Med. Surg..

[39] Wojciechowska J.I., Hewson C.J., Stryhn H., Guy N.C., Patronek G.J., Timmons V. (2005). Evaluation of a questionnaire regarding nonphysical aspects of quality of life in sick and healthy dogs. Am. J. Vet. Res..

