# Supplementary material for: Assessing the Quality of Life of Dogs with Inflammatory Bowel Disease and Their Owners
Source: Vet Sci. 2023 Jun 21;10(7):405. doi: 10.3390/vetsci10070405 (PMC10385821; doi:10.3390/vetsci10070405)
Supplement: Supplementary file 1 [file vetsci-10-00405-s001.zip › Supplementary data. Quality of life questionnaire - english.pdf]

UNIVERSIDAD COMPLUTENSE DE MADRID
FACULTAD DE VETERINARIA
DEPARTAMENTO DE MEDICINA Y CIRUGÍA ANIMAL

Ciudad Universitaria, s/n
28040 Madrid

Dear owner: we request your collaboration to obtain data for the end-of-degree project entitled "Canine inflammatory bowel disease: effect on the quality of life of the dog and its owner". The end-of-degree project constitute a teaching project that students must carry out to be able to finish their studies at the Veterinary College. In the survey, you will be told which of the questions you can answer multiple options simultaneously if you wish. The duration is approximately 5 minutes.

Each answer obtained will be of great importance for our students to reach their final teaching goal, so we deeply thank you for your collaboration. All data obtained with this survey will be exclusively used for teaching and research purposes, always preserving the confidentiality in case of requesting personal data. No transfers or communications of data are provided, except those established by law. You can exercise the rights of access, rectification, or deletion of your data; for more information you can consult our Privacy Policy https://www.ucm.es/como-ejercer-los-derechos

This questionnaire is exclusively aimed at owners of healthy dogs or dogs with inflammatory bowel disease. Indicate if your dog belongs to one of these two groups:

- o Yes
- o No

Continue if your answer to the previous question is affirmative. If it is negative, your survey has been completed. Thank you very much for your collaboration.

To what group does your dog belong?

- o Healthy dogs (without any known disease and without any clinical signs)
- o Dogs diagnosed with inflammatory bowel disease

DATA OF THE DOG

Now let's focus on the information about your dog:

- Age (years)
- Sex (male or female)
- Body weight (kilograms)
- Breed

QUALITY OF LIFE OF THE DOG

In this section, you will refer to how your dog was at the time of the diagnosis of the disease.

Please evaluate the following aspects to know your dog's quality of life at that time:

1. How do you evaluate the general quality of life of your dog? (1: very poor; 10: very good)

   1 2 3 4 5 6 7 8 9 10

2. How do you evaluate the health state of your dog? (1: severely ill; 10: does not seem ill)

   1 2 3 4 5 6 7 8 9 10

3. How do you evaluate the level of activity of your dog (e.g., motivation, curiosity, display of normal behavior…)? (1: aphatetic, lethargic; 10: active)

   1 2 3 4 5 6 7 8 9 10

4. How do you evaluate the interaction of your dog with the owner/family? (1: deteriorated; 10: very good)

   1 2 3 4 5 6 7 8 9 10

5. How do you evaluate the level of stimulation for your dog (e.g., walks, play, training…)? (1: none; 10: many)

   1 2 3 4 5 6 7 8 9 10

OWNER DEMOGRAPHICS

At this time, let's focus on yourself, as the owner:

- Age (years)
- Gender (woman or man)

QUALITY OF LIFE OF THE OWNER

This section is oriented to know a little more about your own quality of life, fundamentally since your dog is ill.

Please value the following items:

1. How do you evaluate your own general quality of life? (1: very poor; 10: very good)

   1 2 3 4 5 6 7 8 9 10

2. Do you think your dog's quality of life can affect your own quality of life? (1: none; 10: too much)

   1 2 3 4 5 6 7 8 9 10

3. Has your dog limited your social life, free time, or day-to-day activities? (1: none; 10: always)

   1 2 3 4 5 6 7 8 9 10

4. How often do you feel burdened by the care of your dog? (1: never; 10: always)

   1 2 3 4 5 6 7 8 9 10

DOG-OWNER RELATIONSHIP

Using a scale from 0 to 10, where 1 is absolutely agree and 10 is totally agree, answer the following questions about the relationship you have with your dog:

1. Do you consider your dog like a child?

   1 2 3 4 5 6 7 8 9 10

2. Do you think your dog understands your mood and your problems?

   1 2 3 4 5 6 7 8 9 10

3. Do you feel closer to your dog than to your friends or family members?

   1 2 3 4 5 6 7 8 9 10

Go to the next and last section only if you are the owner of a dog with inflammatory bowel disease. If your dog is healthy, you have completed the survey. Thank you very much for your collaboration.

IMPACT OF THE DISEASE (only for owners of inflammatory bowel disease dogs)

1. What impact has your dog's disease had on your own quality of life? (1: negative; 10: positive)

   1 2 3 4 5 6 7 8 9 10

2. Has the care of your dog since the diagnosis changed your relationship with him? (1: weaker; 10: stronger)

   1 2 3 4 5 6 7 8 9 10
