# Supplementary material for: Assessing the Quality of Life of Dogs with Inflammatory Bowel Disease and Their Owners
Source: Vet Sci. 2023 Jun 21;10(7):405. doi: 10.3390/vetsci10070405 (PMC10385821; doi:10.3390/vetsci10070405)
Supplement: Supplementary file 1 [file vetsci-10-00405-s001.zip › Supplementary data. Quality of life questionnaire - spanish.pdf]

UNIVERSIDAD COMPLUTENSE DE MADRID
FACULTAD DE VETERINARIA
DEPARTAMENTO DE MEDICINA Y CIRUGÍA ANIMAL

Ciudad Universitaria, s/n
28040 Madrid

Estimado propietario: solicitamos su colaboración para obtener datos para el Trabajo Fin de Grado (TFG) titulado "Enfermedad inflamatoria crónica intestinal canina: efecto sobre la calidad de vida del perro y de su propietario". Los TFG constituyen un proyecto docente que los estudiantes tienen que realizar para poder finalizar sus estudios en el Grado en Veterinaria. A lo largo de la encuesta, se le indicará en cuáles de las preguntas puede contestar varias opciones simultáneamente si así lo desea. La duración es aproximadamente 5 minutos.

Cada respuesta obtenida será de gran importancia para que nuestros estudiantes alcancen su último objetivo docente, por lo que agradecemos profundamente su colaboración. Todos los datos obtenidos con esta encuesta serán empleados, con fines exclusivamente docentes e investigadores, en dicho TFG o en publicaciones que puedan derivar del mismo, preservándose, en todo momento, la confidencialidad en caso de solicitar datos personales. No se prevén cesiones o comunicaciones de datos, salvo las establecidas legalmente. Puede usted ejercer los derechos de acceso, rectificación o supresión de sus datos; para más información puede consultar nuestra Política de Privacidad https://www.ucm.es/como-ejercer-los-derechos

Este cuestionario está dirigido exclusivamente a propietarios de perros sanos o de perros con enfermedad inflamatoria crónica intestinal. Indique si su perro pertenece a uno de estos dos grupos:

- Sí
- No

Continúe si su repuesta a la pregunta anterior es afirmativa. En caso de que sea negativa, ha finalizado su encuesta. Muchas gracias por su colaboración.

¿A qué grupo pertenece su perro?

- Perros sanos (sin ninguna enfermedad conocida y sin ningún signo clínico)
- Perros diagnosticados de enfermedad inflamatoria crónica intestinal

DATOS DEL PERRO

Ahora nos vamos a centrar en la información sobre su perro:

1. Edad (en años)
2. Sexo (hembra/macho)
3. Peso (en kilogramos)
4. Raza

CALIDAD DE VIDA DEL PERRO

En este apartado, se hará referencia a cómo estaba su perro en el momento del diagnóstico de la enfermedad.

Por favor, valore los siguientes aspectos para conocer la calidad de vida de su perro en ese momento:

1. ¿Cómo valoraría en general la calidad de vida de su perro? (donde 1 es muy baja y 10 muy buena)

   1 2 3 4 5 6 7 8 9 10

2. ¿Cómo valoraría el estado de salud de su perro? (donde 1 es gravemente enfermo y 10 no parece enfermo)

   1 2 3 4 5 6 7 8 9 10

3. ¿Cómo valoraría el nivel de actividad de su perro (p. ej. motivación, curiosidad, comportamiento normal…)? (donde 1 es apático, desanimado y 10 es activo)

   1 2 3 4 5 6 7 8 9 10

4. ¿Cómo valoraría la interacción de su perro con el dueño o la familia? (donde 1 es deteriorada y 10 muy buena)

   1 2 3 4 5 6 7 8 9 10

5. ¿Cómo valoraría el nivel de estimulación de su perro (p. ej. paseos, juego, entrenamiento…)? (donde 1 es ninguno y 10 elevado)

   1 2 3 4 5 6 7 8 9 10

DATOS DEMOGRÁFICOS DEL PROPIETARIO

Ahora, nos vamos a centrar en usted mismo, como propietario:

1. Edad (en años)
2. Sexo (hombre/mujer)

CALIDAD DE VIDA DEL PROPIETARIO

Este apartado, está orientado a conocer algo más acerca de su propia calidad de vida, fundamentalmente desde que su perro está enfermo.

Por favor, valore los siguientes aspectos:

1. ¿Cómo califica en general su calidad de vida? (donde 1 es muy baja y 10 muy buena)

   1 2 3 4 5 6 7 8 9 10

2. ¿Considera que la calidad de vida de su perro puede afectar a su propia calidad de vida? (donde 1 es en absoluto de acuerdo y 10 totalmente de acuerdo)

   1 2 3 4 5 6 7 8 9 10

3. ¿Su perro limita o afecta a su vida social, su tiempo libre o sus actividades diarias (trabajo o estudios)? (donde 1 es nada y 10 es siempre)

   1 2 3 4 5 6 7 8 9 10

4. ¿Con qué frecuencia se siente agobiado por el cuidado de su perro? (donde 1 es nunca y 10 es siempre)

   1 2 3 4 5 6 7 8 9 10

RELACIÓN PERRO-DUEÑO

Utilizando una escala de 0 a 10, donde 1 es en absoluto de acuerdo y 10 totalmente de acuerdo, conteste las siguientes preguntas acerca de la relación que tiene con su perro:

1. ¿Considera a su perro como a un hijo?

   1 2 3 4 5 6 7 8 9 10

2. ¿Cree que su perro entiende su estado de ánimo o sus problemas?

   1 2 3 4 5 6 7 8 9 10

3. ¿Se siente más cercano a su perro que a sus amigos o a miembros de la familia?

   1 2 3 4 5 6 7 8 9 10

Pase a la siguiente y última sección solo en el caso de ser propietario de un perro con enfermedad inflamatoria crónica intestinal. Si su perro está sano, ha finalizado la encuesta. Muchas gracias por su colaboración.

IMPACTO DE LA ENFERMEDAD (sólo para propietarios de perros con enfermedad inflamatoria crónica intestinal)

1. ¿Qué impacto ha tenido la enfermedad inflamatoria crónica intestinal de su perro en su propia calidad de vida? (donde 1 es un impacto negativo y 10 un impacto positivo)

   1 2 3 4 5 6 7 8 9 10

2. ¿El cuidado de su perro desde que tiene enfermedad inflamatoria crónica intestinal ha modificado su relación con él? (donde 1 indica que se ha hecho más débil y 10 más fuerte)

   1 2 3 4 5 6 7 8 9 10
